# Association Between Late-Life Weight Change and Dementia: A Population-based Cohort Study

**DOI:** 10.1093/gerona/glac157

**Published:** 2022-08-03

**Authors:** Jie Guo, Anna Marseglia, Ying Shang, Abigail Dove, Giulia Grande, Laura Fratiglioni, Weili Xu

**Affiliations:** Aging Research Center, Department of Neurobiology, Care Sciences and Society, Karolinska Institutet and Stockholm University, Stockholm, Sweden; Division of Clinical Geriatrics, Center for Alzheimer Research, Department of Neurobiology, Care Sciences and Society, Karolinska Institutet, Stockholm, Sweden; Aging Research Center, Department of Neurobiology, Care Sciences and Society, Karolinska Institutet and Stockholm University, Stockholm, Sweden; Department of Medicine, Huddinge, Karolinska Institutet, Stockholm, Sweden; Aging Research Center, Department of Neurobiology, Care Sciences and Society, Karolinska Institutet and Stockholm University, Stockholm, Sweden; Aging Research Center, Department of Neurobiology, Care Sciences and Society, Karolinska Institutet and Stockholm University, Stockholm, Sweden; Aging Research Center, Department of Neurobiology, Care Sciences and Society, Karolinska Institutet and Stockholm University, Stockholm, Sweden; Stockholm Gerontology Research Center, Stockholm, Sweden; Aging Research Center, Department of Neurobiology, Care Sciences and Society, Karolinska Institutet and Stockholm University, Stockholm, Sweden

**Keywords:** APOE, Body mass index, Dementia, Weight gain, Weight loss

## Abstract

**Background:**

The impact of late-life weight changes on incident dementia is unclear. We aimed to investigate the associations of body mass index (BMI) and weight changes with dementia and to explore the role of *APOE* ɛ4 in these associations.

**Methods:**

A total of 1 673 dementia-free participants aged ≥60 and older were followed for an initial 6 years to detect changes in BMI/weight and then for an additional 6 years to detect incident dementia. BMI change ([BMI_first 6-year follow-up_ − BMI_baseline_]/BMI_baseline_) was categorized as stable (≤5%), and moderate (5%–10%) or large (>10%) gain or loss. Weight change (weight_first 6-year follow-up_ − weight_baseline_) was categorized as stable (≤2.5 kg), and moderate (2.5–7.5 kg) or large (>7.5 kg) gain or loss. Dementia was diagnosed following standard criteria. Data were analyzed using Cox regression models.

**Results:**

Over the second 6-year follow-up period, 102 incident dementia cases were identified. Compared with stable BMI, the hazard ratios (95% CI) of dementia were 2.61 (1.09−5.54) and 2.93 (1.72−4.91) for BMI gain or loss >10%, respectively. The risk of dementia was higher among *APOE* ɛ4 carriers experiencing a large BMI gain (9.93 [3.49−24.6]) or loss (6.66 [2.83−14.4]) than *APOE* ɛ4 noncarriers with stable BMI. Similar results were observed for weight change and dementia associations.

**Conclusions:**

BMI and weight changes showed U-shaped associations with dementia risk. Large bodyweight gain and loss alike are associated with an almost 3-fold higher risk of dementia, which may be amplified by *APOE* ɛ4.

Midlife obesity, commonly defined as body mass index (BMI) ≥30 kg/m^2^, is one of the major modifiable risk factors for dementia and contributes to an approximately 33% higher risk of dementia compared with normal BMI (1). The 2020 Lancet Commission on Dementia Prevention and Care highlighted obesity in midlife as a major risk factor for dementia, accounting for about 1% (~500,000) of dementia cases worldwide (2).

BMI is dynamic throughout life, increasing with age during midlife and then stabilizing or declining after age 60 (3). A number of previous studies have investigated the link between late-life BMI and dementia, showing mixed results (4–6). While some studies have identified late-life overweight/obesity as a risk factor for dementia (4,7), most others have reported no significant association between overweight/obesity and dementia (6) or a reduced risk of dementia among older adults with overweight compared with those with normal BMI (20–25 kg/m^2^) (5,8). A shortcoming of these studies is that they examined dementia risk only in relation to BMI at a single time-point, whereas changes in BMI across late life may be more informative (9). Dementia is preceded by a 10–20-year preclinical and prodromal phase during which weight loss may occur (10,11) because of reduced appetite and impaired olfactory function concomitant with neurodegenerative processes (12). Moreover, in terms of biological mechanism, weight gain is associated with an increased risk of vascular diseases, which are well-established risk factors for subsequent dementia in older adults (13). Several studies have investigated the association between weight change in late life and dementia, but with inconsistent results (14–19). Moreover, previous findings may be statistically unreliable due to small sample sizes, limited follow-up time, or methodological issues, such as inappropriately assuming linear associations between weight change and dementia. Thus, to better understand the association between body weight and dementia, a study with a large-scale population, a relatively long follow-up time, and an appropriate nonlinear methodology is warranted.

Apolipoprotein E (APOE) is a protein involved in cholesterol metabolism and lipid homeostasis in the body. The *APOE* ε4 allele has been identified as a major genetic risk factor for Alzheimer’s dementia (AD) (20) and could exacerbate obesity’s impact on cognitive function (21). BMI trajectories in late life also varied by *APOE* genotype, with an earlier and faster decline observed among *APOE* ε4 carriers (22). However, the role of *APOE* ε4 in the association between weight change and dementia risk is unknown.

We hypothesize that adults who both gain and lose weight will be more likely to develop dementia compared with those with stable weight, and the presence of the *APOE* ε4 allele may worsen the impact of weight change. In this population-based cohort study of Swedish older adults, we aimed to (a) investigate the associations between BMI/weight changes and dementia risk and (b) assess whether *APOE* ε4 carrier status modulates these associations.

## Method

### Study Population

The study population was derived from the ongoing population-based Swedish National Study on Aging and Care-Kungsholmen (SNAC-K), including 3 363 participants aged ≥60 and older living at home or in institutions in the Kungsholmen district of central Stockholm at baseline (March 2001 to June 2004). Follow-up assessments were performed every 6 years for the younger age cohorts (60, 66, and 72 years) and every 3 years for the older age cohorts (aged 78+ years), given the higher attrition and more rapid changes in health among older participants.

**Figure 1.**
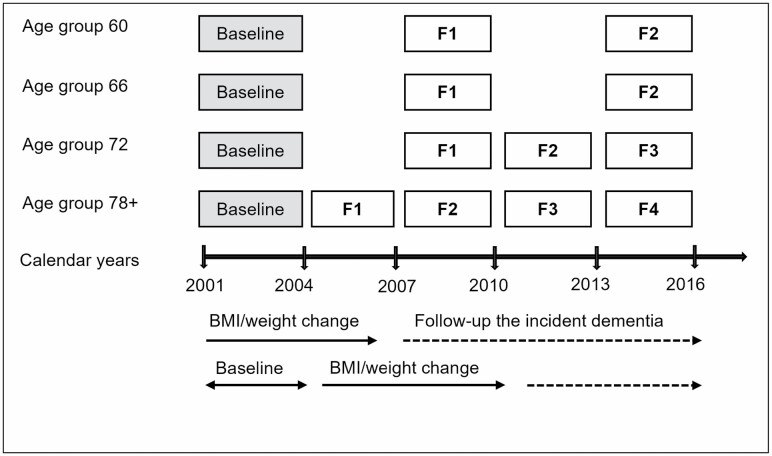
Study timeline for baseline, BMI/weight change, and the dementia follow-up assessment. F1, first follow-up assessment; F2, second follow-up assessment; F3, third follow-up assessment; F4, fourth follow-up assessment.

We detected changes in BMI and weight using measurements at baseline and the first 6-year follow-up (ie, 2001–2004 and 2007–2010) and detected incident dementia during additional 6 years follow-up (ie, from 2007–2010 to 2013–2016; Figure 1). Of all the participants, we excluded 401 participants with prevalent dementia at baseline (*n* = 240) and incident dementia cases during the first 6 years (*n* = 161), leaving 2 962 dementia-free participants. We further excluded 1 104 participants with missing BMI at baseline or the first 6-year follow-up (ie, 123 missing BMI at baseline, 120 missing BMI in the first 6-year follow-up assessment, 515 dead and 346 dropped out within the first 6-year follow-up). Moreover, we excluded 185 who did not participate in the second 6-year follow-up. Therefore, the current study included 1 673 participants (Supplementary Figure 1).

### Standard Protocol Approvals, Registrations, and Patient Consents

Each wave of the SNAC-K data collection was approved by the Karolinska Institutet Ethical Committee and the Regional Ethical Review Board in Stockholm, Sweden. Each participant (or a proxy—such as a close family member—in case of severe cognitive impairment) provided written informed consent at baseline.

### Data Collection

At each wave, data on sociodemographic factors, lifestyle factors, current medication use, and medical conditions were collected by trained staff (nurses and physicians) following a structured protocol (available at http://www.snac-k.se). Additionally, peripheral blood samples were collected for laboratory tests. Data on medical conditions were also ascertained from the Swedish National Patient Register using the International Classification of Disease 10th version codes.

Educational attainment was categorized as an elementary, professional school, high school, or university. Smoking status was classified as never, former, or current smoker. Alcohol consumption was categorized as no/occasional, light-to-moderate (1–14 drinks per week for men or 1–7 drinks per week for women), or heavy (>14 drinks per week for men or >7 drinks per week for women) drinking (23). Physical activity was assessed based on the frequency and intensity of physical exercise and dichotomized as inactive (light-to-intense exercise ≤2–3 times/month) or active (light-to-intense exercise several times per week or every day) (24). Global cognitive function was assessed with the Mini-Mental State Examination (MMSE) administered at each wave of assessment in SNAC-K (25). Detailed information about the identification of chronic conditions, including hypertension, type 2 diabetes (hereafter referred to as diabetes), depression, and cardiovascular diseases (CVDs), was shown in Supplementary Table 1. Information about death was extracted from the Swedish Cause of Death Registry (SCDR; June 2001–December 2016).

*APOE* genotypes were assessed using a microsequencing method (AffiGen *APOE*, Sangtec Medical) based on a polymerase chain reaction with biotinylated primers and dichotomized into ε4 carriers and noncarriers.

### Assessment of BMI Change and Weight Change

At each wave, participants’ weights and heights were measured by trained staff using a standard scale while participants were wearing light clothing and no shoes. In the case of missing height and weight measurements, self-reported values were used. BMI (kg/m^2^) was calculated as weight in kilograms divided by squared height in meters and categorized into 4 groups: underweight (<20 kg/m^2^), normal weight (20–25 kg/m^2^), overweight (25–30 kg/m^2^), and obesity (≥30 kg/m^2^) (26). Percent change in BMI was defined as the difference between BMI at the first 6-year follow-up and baseline divided by baseline BMI ([BMI _first 6-year follow-up_ − BMI _baseline_]/BMI _baseline_). Besides the continuous BMI change, we also categorized BMI change into 5 groups. A BMI change ≤5% was considered stable, and BMI loss/gain was categorized into the following groups: large loss (>10%), moderate loss (5%–10%), moderate gain (5%–10%), and large gain (>10%) (27). Similarly, weight change was calculated as the difference between weight at the first 6-year follow-up and weight at baseline (weight _first 6-year follow-up_ − weight _baseline_). We defined weight change ≤2.5 kg as stable and categorized weight loss/gain into the following groups: large loss (>7.5 kg), moderate loss (2.5–7.5 kg), moderate gain (2.5–7.5 kg), and large gain (>7.5 kg).

### Assessment of Dementia

Dementia was diagnosed according to Diagnostic and Statistical Manual of Mental Disorders (*DSM-IV*) criteria using a validated 3-step procedure (28). First, a preliminary diagnosis was made by the examining physician. Second, all dementia diagnoses were independently reviewed by a second physician, who made a second preliminary diagnosis. Third, in case of disagreement between these 2 diagnoses, a deciding diagnosis was sought by a neurologist external to the data collection (29). Standard criteria were used to diagnose AD and vascular dementia (VaD) (30,31). For participants who died during follow-up without a prior dementia diagnosis, dementia status was verified through death certificates and clinical charts via linkage to the SCDR and/or medical records at hospital discharge when available.

### Statistical Analysis

Data were presented as means and standard deviations (*SD*s) for continuous variables or as numbers (percentages) for categorical variables. Baseline characteristics of the study population by incident dementia status were compared using *t*-tests for continuous variables and chi-square tests for categorical variables.

Hazard ratios (HRs) and 95% confidence intervals (CIs) for incident dementia in relation to BMI change were estimated with Cox proportional hazard models, using follow-up time as the timescale. In the current study, follow-up time was calculated as the time from the 6-year follow-up (2007–2010) until dementia diagnosis, death, or last examination, whichever occurred first. The proportional hazard assumption was assessed through the interaction between log(time) and Schoenfeld residuals; no violations of proportionality were observed. The associations of continuous BMI change with subsequent dementia were modeled using restricted cubic splines with 4 knots at the 5th, 35th, 65th, and 95th percentiles (32), with adjustment for potential confounders. Next, we analyzed the associations between BMI change categories and dementia. The basic models were adjusted for age, sex, and education. The multi-adjusted models were further adjusted for smoking, alcohol consumption, physical activity, hypertension, CVDs, diabetes, depression, and *APOE*-ε4. Stratified analyses by *APOE* genotype (ε4 carriers vs noncarriers) were performed. Interactions between *APOE* genotype and BMI change groups were also tested. We further combined *APOE* genotype and BMI change (5 groups: large loss, moderate loss, stable, moderate gain, and large gain) and explored their joint effects on dementia. We also conducted the aforementioned analyses with weight change as the exposure, and we additionally adjusted BMI at baseline in the multi-adjusted models.

Statistical analyses were performed using SAS 9.4 (SAS Institute, Cary, NC). All *p*-values were 2-sided, and we defined statistical significance as *p* < .05.

### Supplementary Analyses

We compared the baseline characteristics of our analytical population (*n* = 1 673) with the entire SNAC-K population (*n* = 3 363). We compared the baseline characteristics of the study population across the different BMI/weight change categories. We further explored the associations between BMI/weight change and dementia subtypes (ie, AD and VaD). To address the relationship between weight/BMI change category and cognitive trajectories before dementia diagnosis, we used linear mixed-effect models to estimate the annual changes in MMSE during the first 6 years of follow-up. In further sensitivity analysis, we repeated the results after excluding participants with cerebrovascular disease. We also repeated the analyses using imputed BMI/weight for participants with the second 6-year follow-up information but missing BMI/weight at baseline (*n* = 11) or the 6-year follow-up assessment (*n* = 49). Finally, the restricted cubic spline analysis was repeated using a different set of 4 knots as the cutoffs corresponding to the cutoffs to categorize bodyweight change (ie, −10%, −5%, 5%, and 10% for percent change in BMI and −7.5, −2.5, 2.5, and 7.5 kg for weight change).

## Results

### Baseline Characteristics of the Study Population

Among the 1 673 dementia-free participants, the mean age (*SD*) at baseline was 69.4 (8.7) years and 1 029 were female (61.5%). Table 1 shows the baseline characteristics of participants by incident dementia. Compared with participants without incident dementia, those with incident dementia were older, less educated, less engaged in physical activity, and more likely to have CVDs, hypertension, a lower MMSE score, or at least one copy of the *APOE* ɛ4 allele. Moreover, participants with incident dementia were more likely to experience a large loss or gain in BMI/weight (Table 1).

**Table 1.** Characteristics of Study Population by Incident Dementia During the Second 6-y Follow-up (*N* = 1 673)

Characteristics	Total (*n* = 1 673)	Nondementia (*n* = 1 571)	Dementia (*n* = 102)	*p*-Value
Baseline				
Age, y	69.4 ± 8.7	69.0 ± 8.6	75.9 ± 7.4	<.001
Age cohorts				<.001
60/66	912 (54.5)	897 (57.1)	15 (14.7)	
72/78	519 (31.0)	465 (29.6)	54 (52.9)	
81−87	205 (12.3)	174 (11.1)	31 (30.4)	
90+	37 (2.2)	35 (2.2)	2 (2.0)	
Female	1 029 (61.5)	972 (61.9)	57 (55.9)	.228
Education level				.008
Elementary	178 (10.6)	162 (10.3)	16 (15.7)	
Professional school	657 (39.3)	606 (38.6)	51 (50.0)	
High school	182 (10.9)	172 (10.9)	10 (9.8)	
University	655 (39.2)	630 (40.1)	25 (24.5)	
Smoking status				.907
Never	758 (45.3)	710 (45.2)	48 (47.1)	
Former	688 (41.1)	648 (41.2)	40 (39.2)	
Current	220 (13.2)	206 (13.1)	14 (13.7)	
Alcohol consumption				.046
No or occasional	403 (24.1)	368 (23.4)	35 (34.3)	
Light-to-moderate	959 (57.3)	909 (57.9)	50 (49.0)	
Heavy drinking	307 (18.4)	290 (18.5)	17 (16.7)	
Physical activity				.013
Inactive	319 (19.1)	290 (18.5)	29 (28.4)	
Active	1 354 (80.9)	1 281 (81.5)	73 (71.6)	
Cardiovascular diseases	319 (19.1)	288 (18.3)	31 (30.4)	.003
Cerebrovascular disease	67 (4.0)	59 (3.8)	8 (7.8)	.041
Ischemic heart disease	178 (10.6)	160 (10.2)	18 (17.6)	.018
Heart failure	75 (4.5)	71 (4.5)	4 (3.9)	.777
Atrial fibrillation	89 (5.3)	82 (5.2)	7 (6.9)	.474
Other cardiovascular disease	28 (1.7)	26 (1.7)	2 (2.0)	.816
Hypertension	1 197 (71.5)	1 115 (71.0)	82 (80.4)	.041
Diabetes	118 (7.1)	106 (6.7)	12 (11.8)	.057
Depression	124 (7.4)	113 (7.2)	11 (10.8)	.180
*APOE* ɛ4 carrier	462 (27.6)	417 (26.5)	45 (44.1)	<.001
MMSE	29.2 ± 1.0	29.2 ± 1.0	28.6 ± 1.3	<.001
Baseline BMI, kg/m^2^	26.0 ± 3.8	26.0 ± 3.8	25.8 ± 4.3	.703
Baseline BMI				.115
Underweight (<20)	46 (2.7)	40 (2.5)	6 (5.9)	
Normal (20–25)	687 (41.1)	644 (41.0)	43 (42.2)	
Overweight (25–30)	711 (42.5)	675 (43.0)	36 (35.3)	
Obese (≥30)	229 (13.7)	212 (13.5)	17 (16.7)	
During the initial 6-y follow-up				
BMI change				<.001
Large loss (>10%)	180 (10.8)	154 (9.8)	26 (25.5)	
Moderate loss (5%–10%)	263 (15.7)	244 (15.5)	19 (18.6)	
Stable (≤5%)	972 (58.1)	928 (59.1)	44 (43.1)	
Moderate gain (5%–10%)	173 (10.3)	168 (10.7)	5 (4.9)	
Large gain (>10%)	85 (5.1)	77 (4.9)	8 (7.8)	
Weight change				<.001
Large loss(>7.5 kg)	180 (10.8)	154 (9.8)	26 (25.5)	
Moderate loss (2.5–7.5 kg)	429 (25.6)	400 (25.5)	29 (28.4)	
Stable (≤2.5 kg)	752 (44.9)	719 (45.8)	33 (32.4)	
Moderate gain (2.5–7.5 kg)	241 (14.4)	234 (14.9)	7 (6.9)	
Large gain(>7.5 kg)	71 (4.2)	64 (4.1)	7 (6.9)	

*Notes: APOE* ɛ4 = apolipoprotein ɛ4 allele; BMI = body mass index; MMSE = Mini-Mental State Examination. Data are presented as means ± standard deviations for continuous variables or number (proportion %) for categorical variables.

Missing data: 1 for education, 7 for smoking status, 4 for alcohol consumption, 5 for diabetes, and 28 for *APOE* ɛ4.

Compared with participants with stable BMI, those with large BMI loss were older, less educated, less engaged in active physical activity, and more likely to have CVDs, hypertension, and depression, or a lower MMSE score, while participants with large BMI gain were younger and more likely to be current smokers (Supplementary Table 2). Similar distributions of characteristics were also observed across weight change categories (Supplementary Table 3).

### Risk of Dementia According to BMI/Weight Change

**Figure 2.**
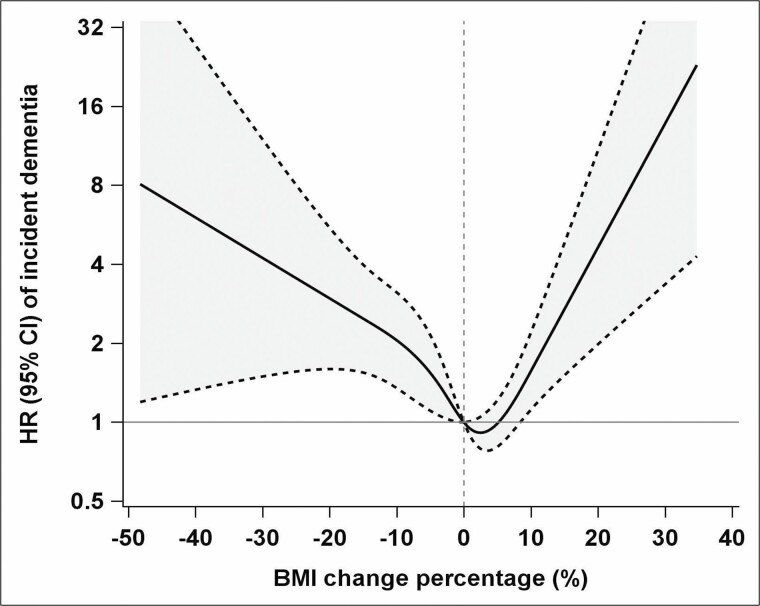
Associations of body mass index (BMI) change over 6 years with the risk of incident dementia. Hazard ratios (HRs) and 95% confidence intervals (CIs) were calculated using multivariable Cox regression models with restricted cubic splines. Risk estimates were adjusted for baseline age, sex, education, smoking status, alcohol consumption, physical activity, medical history of cardiovascular diseases (ischemic heart disease, heart failure, atrial fibrillation, cerebrovascular disease, other cardiovascular diseases), hypertension, diabetes, depression, and *APOE* ɛ4. *p* values for overall association and *p* values for nonlinear association were both <.05.

During a median of 5.78 years of follow-up (interquartile range: 5.39−5.94 years), 102 participants developed dementia. Figure 2 illustrates the U-shaped associations between BMI change and incident dementia; those with stable BMI had the lowest risk of dementia. Among participants with BMI loss <0%, the HR per 1-unit loss in BMI was 1.06 (95% CI 1.01–1.10). Among those with BMI gain >0%, the HR per 1-unit gain in BMI was 1.07 (95% CI 1.00–1.14). Compared with participants with stable BMI, the risk of dementia increased significantly in those with BMI loss >10% (multi-adjusted HR = 2.93, 95% CI 1.72–4.91) or gain >10% (HR = 2.61, 95% CI 1.09–5.54; Table 2). The U-shaped associations were also observed for weight change and dementia (Supplementary Figure 2 and Supplementary Table 4).

**Table 2.** HRs and 95% CIs of the Association of BMI Change Over 6 y With Dementia Risk

BMI Change ^a^	Number of Subjects	Number of Events/Person-years	HR (95% CI)	
			Basic-adjusted ^b^	Multi-adjusted ^c^
BMI change (continuous)				
Loss <0%, per 1-unit decrease	924	72/4624	**1.05 (1.02−1.09)**	**1.06 (1.01−1.10)**
Gain >0%, per 1-unit increase	696	29/3735	**1.09 (1.02−1.14)**	**1.07 (1.004−1.14)**
BMI change (categorical)				
Large loss (>10%)	180	26/756	**3.04 (1.80−5.05)**	**2.93 (1.72−4.91)**
Moderate loss (5−10%)	263	19/1367	1.22 (0.69−2.08)	1.06 (0.60−1.83)
Stable (≤5%)	972	44/5133	Reference	Reference
Moderate gain (5−10%)	173	5/954	0.73 (0.25−1.69)	0.79 (0.27−1.83)
Large gain (>10%)	85	8/431	**2.61 (1.12−5.35)**	**2.61 (1.09−5.54)**

*Notes:* CI = confidence interval; HR = hazard ratio. Bold values are statistically significant (*p*-Value <.05).

^a^Percent change in BMI = [BMI _6th year follow-up_ − BMI _baseline_]/BMI_baseline_.

^b^Adjusted for age, sex, and education.

^c^Additionally adjusted for smoking status, alcohol consumption, physical activity, hypertension, cardiovascular diseases, diabetes, depression, and *APOE* ɛ4.

Besides adjusting for baseline BMI in the weight change-dementia analysis, we also conducted stratified analysis by baseline BMI (<25 and ≥25 kg/m^2^). Among participants with baseline BMI ≥25 kg/m^2^, undergoing a large weight loss or gain was associated with increased dementia risk compared with having a stable weight. Among participants with baseline BMI <25 kg/m^2^, large weight loss was still significantly associated with increased dementia risk, while the association between large weight gain and dementia was not statistically significant, partly due to the small sample size in this group. There was no statistically significant interaction between baseline BMI groups and weight change for dementia (*p* for interaction = .334; Supplementary Table 5).

### Joint Effects of *APOE*-ε4 and BMI/Weight Change on Dementia

**Figure 3.**
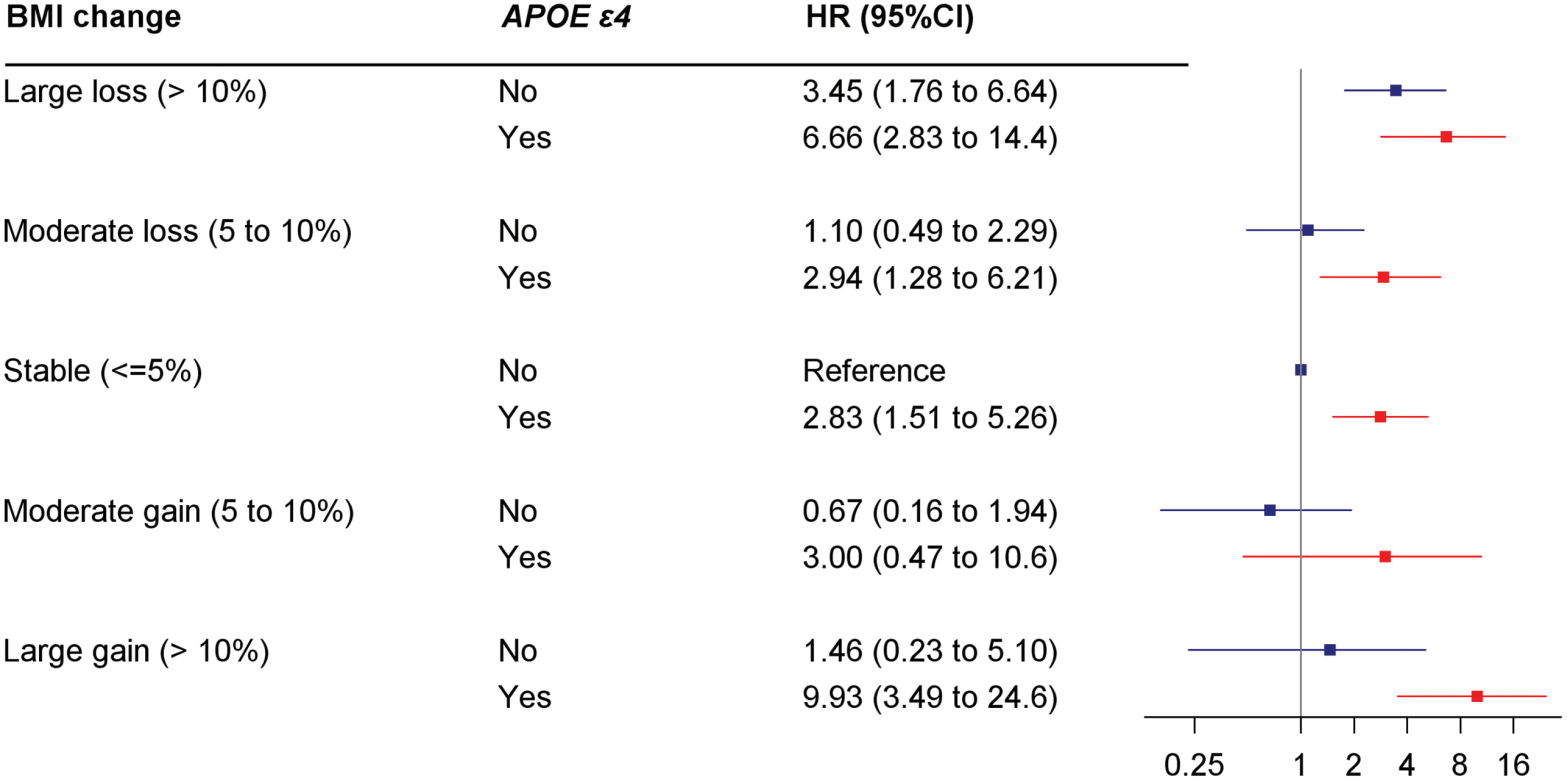
Hazard ratios (HRs) and 95% confidence intervals (CIs) of the joint effect of APOE ɛ4 and body mass index (BMI) change on incident dementia. Adjusted for age, sex, education, smoking status, alcohol consumption, physical activity, medical history of cardiovascular diseases, hypertension, diabetes, and depression.

Figure 3 shows the joint effect of *APOE* ɛ4 and BMI change on the risk of dementia. Compared with *APOE* ɛ4 noncarriers with stable BMI, *APOE* ɛ4 carriers with large BMI loss (HR = 6.66, 95% CI 2.83−14.4) or gain (HR = 9.93, 95% CI 3.49–24.6) had higher dementia risk. Moreover, among participants with BMI gain >10%, *APOE* ɛ4 carriers were associated with a higher risk of dementia compared with noncarriers (HR = 6.81, 95% CI 1.52–47.4). Among those with BMI loss >10%, *APOE* ɛ4 carriers also showed a higher risk of dementia compared with noncarriers (HR = 1.93, 95% CI 0.79–4.41, *p* = .129), albeit not statistically significant. Similar results were observed for the combination of *APOE* ɛ4 and weight change on dementia (Supplementary Figure 3). However, the multiplicative interaction between *APOE* ɛ4 and BMI change on dementia risk was not statistically significant (*p* for interaction = .710). Among *APOE* ɛ4 noncarriers, there was a significant risk of dementia with large BMI loss but not large BMI gain. Among *APOE* ɛ4 carriers, those who experienced either large BMI loss or large BMI gain had an increased risk of dementia compared with those with stable BMI (Supplementary Table 6). The impact of large weight loss remained among *APOE* ɛ4 noncarriers, though there was no significant association between large weight change and dementia among *APOE* ɛ4 carriers, partly due to the small sample size (Supplementary Table 7).

### Supplementary Results

Compared with the total SNAC-K population, those included in the present study were younger, more educated, more physically active, less likely to have CVDs and diabetes, and more likely to have a higher MMSE score (results not shown, all *p* values < .05). There were U-shaped associations between BMI/weight changes and both AD and VaD, with a higher risk for participants with large BMI/weight loss or gain (Supplementary Table 8). Linear mixed-effect models showed that large BMI loss or gain was related to a faster decline in MMSE over 6 years compared with stable BMI. Similarly, compared with stable weight, large weight loss or gain was associated with a faster decline in MMSE (*p* < .05; Supplementary Table 9). After excluding participants with cerebrovascular disease, the large BMI/weight loss remained associated with an increased risk of dementia. Large BMI gain was no longer statistically significant associated with dementia (HR = 2.06, 95% CI 0.81–4.60, *p* = .098), though the impact of large weight gain remained (Supplementary Table 10). The associations between body weight change and dementia were not largely altered after imputing missing data on body weight (Supplementary Table 11). Finally, the curves for the dose–response associations between BMI/weight change and dementia did not change largely with 4 knots corresponding to the cutoffs used to categorize bodyweight change compared with 4 default knots at the 5th, 35th, 65th, and 95th percentiles (Supplementary Figure 4).

## Discussion

In this long-term population-based longitudinal cohort of dementia-free older adults, we found that (a) BMI/weight change over 6 years had a U-shaped association with incident dementia; (b) large BMI/weight loss and gain alike were related to increased dementia risk after adjustment for potential confounders; and (3) the risk of dementia was especially high for *APOE* ɛ4 carriers who experience large BMI/weight loss or gain.

Previous studies that have explored the association between weight change in late life and dementia mostly observed an increased dementia risk linked to weight loss (14–16,19,27,33,34). Conversely, in some studies, weight gain has been either not significantly related to dementia or seemed to be with reduced dementia risk (15,19,35). A recent meta-analysis pooling data from 19 prospective cohorts and 4 clinical trials showed that older adults (aged ≥60 and older) with ≥0.5% annual weight loss had approximately one-third greater risk of all-cause dementia than those with a relatively stable weight. However, a weight gain of 0.5% or above was not associated with increased dementia risk (33). Similarly, in an Australian cohort of 4 181 men aged 65–84 years, men whose BMI fell >1 kg/m^2^ over 5 years had about 90% higher dementia risk compared with those with a stable BMI (change ≤1 kg/m^2^) (16). Furthermore, a reverse “J-shaped” association was observed (ie, increasing BMI was associated with a slightly higher risk of dementia), although this association was not statistically significant (16).

There are several possible explanations for the discrepancies in previous studies regarding the association between weight gain and dementia. One possibility could be the small sample size (for both exposures and outcomes) (15,16). Moreover, weight gain is hypothesized to act as a risk factor for dementia by activating cardiometabolic pathology pathways, so a shorter follow-up that does not encompass the progression from weight gain to the occurrence of cardiometabolic disorders and to subsequent dementia is not sufficient to detect the risk of dementia. Additionally, it is possible that examining the effect of body weight change on dementia using linear models could mask the increased risk in relation to weight gain. A previous cohort study of 67,219 Koreans aged 60–79 years with a mean of 5.3 years follow-up showed that >10% BMI loss/gain are associated with dementia risk (27). In line with this study, we found that both excess body weight loss and gain (BMI change >10% or weight change >7.5 kg over 6 years) were associated with about 2-fold greater dementia risk than stable body weight among Swedish older adults.

Previous studies have reported that the *APOE* ɛ4 allele can worsen the cognitive function in relation to obesity (21). However, no study to date has examined whether similar effects could also exist in relation to weight gain, not necessarily obesity. In the current study, the lack of significant multiplicative interaction between BMI change and *APOE* genotypes indicated that the strength of associations between BMI change and dementia might not differ by *APOE* genotypes. Maintaining a stable BMI may benefit both *APOE* ɛ4 carriers and noncarriers. Moreover, among individuals experiencing a large BMI change, the presence of the *APOE* ɛ4 allele seemed to have an elevated risk of dementia.

The mechanisms underlying increased dementia risk associated with weight loss or gain remain unclear. Indeed, it has been suggested that weight loss may occur during the preclinical and prodromal phases of dementia (ie, start 10–20 years before the clinical diagnosis of dementia) (10,11). Reduced appetite and impaired olfactory function concomitant with neurodegenerative processes may reduce an individual’s energy intake and lead to weight loss (12). Moreover, as indicated from our supplementary analysis, participants with BMI/weight loss largely experienced a faster decline in cognitive function, which usually preceded the diagnosis of dementia. BMI change and cognitive decline may also share similar mechanisms, like the accumulation of β-amyloid in aging brains (36). In this way, weight loss can be considered as a marker of preclinical and prodromal dementia. Additionally, weight loss could be causally related to dementia through direct biological pathways. For example, weight loss due to loss of fat mass could impact neurological function through changes in the activity of the hormone leptin, which is mainly secreted by adipose tissues but also expressed in the brain (37). Moreover, weight loss due to the loss of muscle mass could involve mechanisms overlapping with dementia, including inflammation, oxidative stress, and hormonal dysregulation (38,39). On the other hand, weight gain due to excessive fat mass could increase the risk of dementia through the same set of pathophysiological mechanisms that underlie other common age-related cardiometabolic disorders, including atherosclerosis, arterial stiffness, insulin resistance, and enhanced secretion of proinflammatory cytokines (40,41). Our study showed that weight gain is related to an increased risk of both AD and VaD, suggesting that weight gain may contribute to dementia risk through both neurodegenerative and vascular processes.

### Strengths and Limitations

Strengths of the study include the large-scale, population-based, longitudinal, study design, which enabled repeated measurements of body weight over a long follow-up period. Moreover, the clinical diagnosis of dementia was made by physicians following standard criteria, thus minimizing the underreporting of dementia compared with studies that only identify dementia using electronic health records. However, some limitations of our study should be acknowledged. First, although BMI was calculated using measured weight and height for a majority of study population, some participants’ BMIs were based on self-reported information (82 and 230 participants had only self-reported BMI at baseline and at the 6-year follow-up assessment, respectively). However, a previous study demonstrated that the correlation between self-reported and measured BMI in SNAC-K was high (Pearson correlation coefficient’s *r* = .95, *p* < .001) (3). Second, we were not able to distinguish between unintentional weight loss and intentional weight loss (eg, due to improved diet and a more active lifestyle), which have opposite impacts on cognitive function (42). This may lead to an underestimation of the impact of unintentional weight loss. Third, due to the limited repeated measurements in this study, we could only explore BMI/weight changes between 2 timepoints rather than BMI/weight fluctuation. Future studies including information on weight variability are needed to provide more evidence on the impact of varied patterns of weight change on dementia. Fourth, the date of dementia onset was defined as the date of dementia diagnosis during the follow-up assessment (ie, every 3 years for older cohorts and 6 years for younger cohorts), even though dementia may have set in at any time during adjacent follow-up assessments. This could contribute to an underestimation of the associations in relation to dementia. Fifth, the joint effect between *APOE* genotype and body weight change should be interpreted with caution given the small sample size of participants who were *APOE* ɛ4 carriers and experienced a large change in body weight. Further large population-based cohort studies are warranted to verify our results. Sixth, we cannot rule out the impact of unmeasured confounders or residual confounding, though our analyses were adjusted for a wide range of covariates. Finally, participants included in the main analyses were healthier than those who were excluded, which may have resulted in an underestimation of the observed associations. Moreover, as the SNAC-K population is urban and has a higher level of education compared with people living in other areas of Sweden, caution is required when generalizing these results to other populations.

## Conclusion

Our study provides evidence that large bodyweight loss and gain alike are associated with an increased risk of dementia among older adults. The risk of dementia with large bodyweight change may be higher for *APOE* ɛ4 carriers than noncarriers. Further studies are warranted to identify factors related to largely weight change and recommendations for preventing large bodyweight loss and gain among older adults should be considered in clinical and public health practices for dementia prevention. Large-scale cohort studies with long-term follow-up are warranted to verify the observed associations in our study.

## Supplementary Material

glac157_suppl_Supplementary_File
