# Supplementary material for: Association Between Late-Life Weight Change and Dementia: A Population-based Cohort Study
Source: J Gerontol A Biol Sci Med Sci. 2022 Aug 3;78(1):143–50. doi: 10.1093/gerona/glac157 (PMC9879755; doi:10.1093/gerona/glac157)
Supplement: glac157_suppl_Supplementary_File [file glac157_suppl_supplementary_file.pdf]

Supplementary file: Association between late-life weight change and dementia: A population-based cohort study

Contents

Supplementary Figure 1. Flow chart of study population... 2

Supplementary Table 2. The baseline characteristics of study population by body mass index (BMI) change (N= 1,673). 4

Supplementary Table 3. The baseline characteristics of study population by weight change (N= 1,673).. 5

Supplementary Figure 2. Associations of weight change over 6 years with the risk of incident dementia.. 6

Supplementary Table 4. Hazard ratios (HRs) and 95% confidence intervals (CIs) of the association of weight change over 6 years with dementia risk.. 7

Supplementary Table 5. Hazard ratios (HR) with 95% confidence intervals (CI) of incident dementia for weight change over 6 years by baseline body mass index (BMI). 8

Supplementary Figure 3. Hazard ratios (HRs) and 95% confidence intervals (CIs) of the joint effect of APOE ε4 and weight change on incident dementia.. 9

Supplementary Table 6. Hazard ratios (HR) with 95% confidence intervals (CI) of incident dementia for body mass index (BMI) change by APOE genotype... 10

Supplementary Table 7. Hazard ratios (HR) with 95% confidence intervals (CI) of incident dementia for weight change by APOE genotype.. 11

Supplementary Table 8. Hazard ratios (HR) and 95% confidence intervals (CI) of incident Alzheimer's disease (AD), vascular dementia (VaD) according to BMI change and weight change over 6 years.. 12

Supplementary Table 9. Mixed effect model's β coefficients and 95% confidence interval (CI) for the annual Mini-Mental State Examination (MMSE) change related to BMI/weight change within the initial 6 years follow-up.. 13

Supplementary Table 10. Hazard ratios (HR) with 95% confidence intervals (CI) of incident dementia according to BMI change and weight change excluding participants with cerebrovascular disease.. 14

Supplementary Table 11. Hazard ratios (HR) and 95% confidence intervals (CI) of the association of BMI/weight change with dementia risk using datasets with imputed BMI or weight.. 15

Supplementary Figure 4. Associations of body mass index (BMI) and weight change with risk of incident dementia.. 16

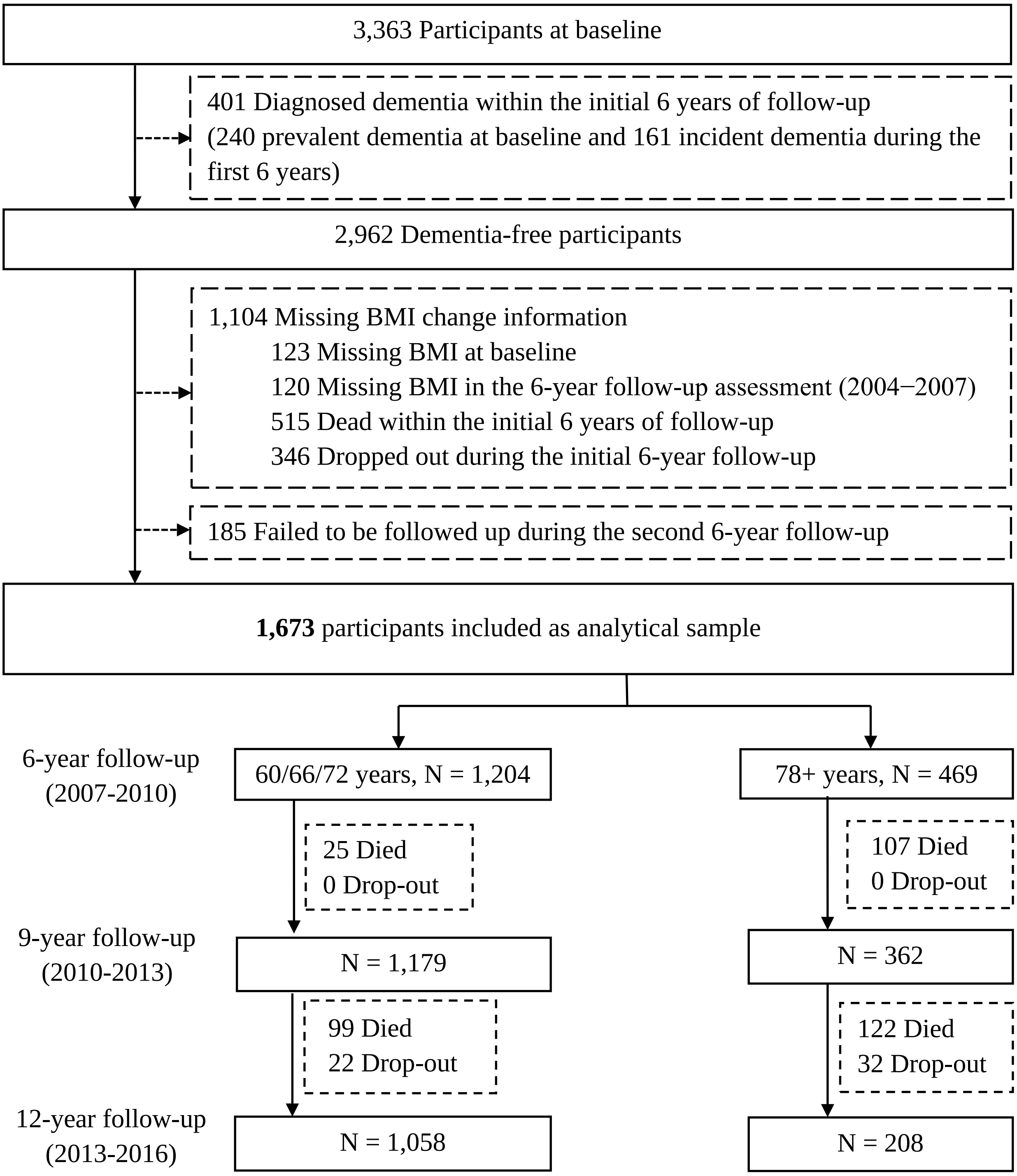

Supplementary Figure 1. Flow chart of study population.

Supplementary Table 1. Description of identifying chronic conditions

**Condition**	**Definition**
Hypertension	Measured systolic and diastolic blood pressure ⩾140/90 mmHg, use of antihypertensive medication, or medical records from the NPR (ICD-10 codes: I10-13, and I15)
Type 2 diabetes	Self-reported medical history, use of glucose-lowering medications, medical records from the NPR (ICD-10 code E11), or glycated hemoglobin ⩾6.5%
Depression	Medical records from the NPR (ICD-10 codes: F30-34, F38-39, and F412), or diagnosed by physicians according to *DSM-IV* in clinical examinations
Cardiovascular diseases	Diagnosed by physicians in clinical examination, electrocardiogram, medication use, or medical records from the NPR (ICD-10 codes as follows)
Ischemic heart disease	ICD-10 codes: I20-22, I24-25, Z951, and Z955
Atrial fibrillation	ICD-10 code: I48
Heart failure	ICD-10 codes: I110, I130, I132, I27, I280, I42–43, I50, I515, I517, I528, Z941, and Z943
Cerebrovascular disease	ICD-10 codes: G45-46, I60-64, I67, and I69
Other cardiovascular diseases	ICD-10 codes: I09, I281, I310-311, I456, I495, I498, I70-72, I790-791, I950, I951, I958, Q20-21, Q24-28, and Z958-959

Notes: NPR, National Patient Registry; ICD-10, International Classification of Disease 10th version; *DSM-IV*, Diagnostic and Statistical Manual of Mental Disorders, 4th edition.

Supplementary Table 2. The baseline characteristics of study population by body mass index (BMI) change (N= 1,673).

Characteristics	BMI change over 6 years				
	**Large loss (> 10%)**	**Moderate loss (5 - 10%)**	**Stable (≤ 5%)**	**Moderate gain (5 - 10%)**	**Large gain (> 10%)**
Number	180 (10.8)	263 (15.7)	972 (58.1)	173 (10.3)	85 (5.1)
Age, years	75.5 ± 8.7*	71.2 ± 8.7*	68.5 ± 8.3	66.3 ± 7.3*	67.6 ± 8.5
Female	129 (71.7)**	168 (63.9)	561 (57.7)	110 (63.6)	61 (71.8)*
Education level					
Elementary/Professional school	121 (67.2)**	139 (53.1)	456 (46.9)	76 (43.9)	43 (50.6)
High school/University	59 (32.8)**	123 (47.0)	516 (53.1)	97 (56.1)	42 (49.4)
Current smoker	26 (14.4)	33 (12.5)	113 (11.6)	27 (15.6)	21 (24.7)*
Heavy drinking	29 (16.1)	44 (16.7)	181 (18.6)	39 (22.5)	14 (16.5)
Active physical activity	126 (70.0)**	214 (81.4)	809 (83.2)	138 (79.8)	67 (78.8)
Cardiovascular diseases	54 (30.0)**	59 (22.4)	169 (17.4)	27 (15.6)	10 (11.8)
Hypertension	152 (84.4)**	188 (71.5)	686 (70.6)	112 (64.7)	59 (69.4)
Diabetes	19 (10.6)	21 (8.0)	65 (6.7)	11 (6.4)	2 (2.4)
Depression	20 (11.1)*	15 (5.7)	61 (6.3)	18 (10.4)	10 (11.8)
APOE ε4 carrier	48 (26.7)	74 (28.1)	281 (28.9)	34 (19.7)*	25 (29.4)
MMSE	28.8 ± 1.3*	29.2 ± 1.0	29.3 ± 1.0	29.3 ± 1.1	29.2 ± 1.1

Notes: APOE ε4, apolipoprotein ε4 allele; MMSE, Mini-Mental State Examination. Data are presented as means ± standard deviations or number (proportion %). We compared the means or proportion of BMI large loss/gain and moderate loss/gain with the stable BMI change. *, P <0.05; **, P <0.001. Abbreviations: *ApoE* ε4, apolipoprotein ε4 allele. Missing data: 1 for education, 7 for smoking status, 4 for alcohol consumption, 5 for diabetes, and 28 for *ApoE* ε4.

Supplementary Table 3. The baseline characteristics of study population by weight change (N= 1,673).

Characteristics	Weight change over 6 years				
	**Large loss (> 7.5 kg)**	**Moderate loss (2.5-7.5 kg)**	**Stable (≤ 2.5 kg)**	**Moderate gain (2.5-7.5 kg)**	**Large gain (> 7.5 kg)**
Number	180 (10.8)	429 (25.6)	752 (44.9)	241 (14.4)	71 (4.2)
Age, years	74.0 ± 9.1*	71.3 ± 8.7*	68.5 ± 8.2	66.2 ± 7.6	66.6 ± 7.7*
Female	115 (63.9)	284 (66.2)*	446 (59.3)	138 (57.3)	46 (64.8)
Education level					
Elementary/ Professional school	109 (60.9)*	228 (53.2)	354 (47.1)	112 (46.5)	32 (45.1)
High school/ University	70 (39.1)*	201 (46.9)*	398 (52.9)	129 (53.5)	39 (54.9)
Current smoker	27 (15.0)	55 (12.8)	86 (11.4)	35 (14.5)	17 (23.9)*
Heavy drinking	24 (13.3)	79 (18.4)	145 (19.3)	49 (20.3)	10 (14.1)
Active physical activity	120 (66.7)*	358 (83.4)	630 (83.8)	192 (79.7)	54 (76.1)
Cardiovascular diseases	56 (31.1)**	97 (22.6)**	114 (15.2)	42 (17.4)	10 (14.1)
Hypertension	153 (85.0)**	323 (75.3)**	512 (68.1)	158 (65.6)	51 (71.8)
Diabetes	22 (12.2)*	37 (8.6)	43 (5.7)	14 (5.8)	2 (2.8)
Depression	17 (9.4)	23 (5.4)	51 (6.8)	25 (10.4)	8 (11.3)
ApoE ε4 carrier	51 (28.3)	123 (28.7)	215 (28.6)	55 (22.8)	18 (25.4)
MMSE	28.9 ± 1.3*	29.2 ± 0.9	29.2 ± 1.0	29.3 ± 1.1	29.2 ± 1.0

Notes: APOE ε4, apolipoprotein ε4 allele; MMSE, Mini-Mental State Examination. Data are presented as means ± standard deviations or number (proportion %). We compared the means or proportion of weight large loss/gain and moderate loss/gain with the stable BMI change. *, P <0.05; **, P <0.001. Abbreviations: *ApoE* ε4, apolipoprotein ε4 allele. Missing data: 1 for education, 7 for smoking status, 4 for alcohol consumption, 5 for diabetes, and 28 for *ApoE* ε4.

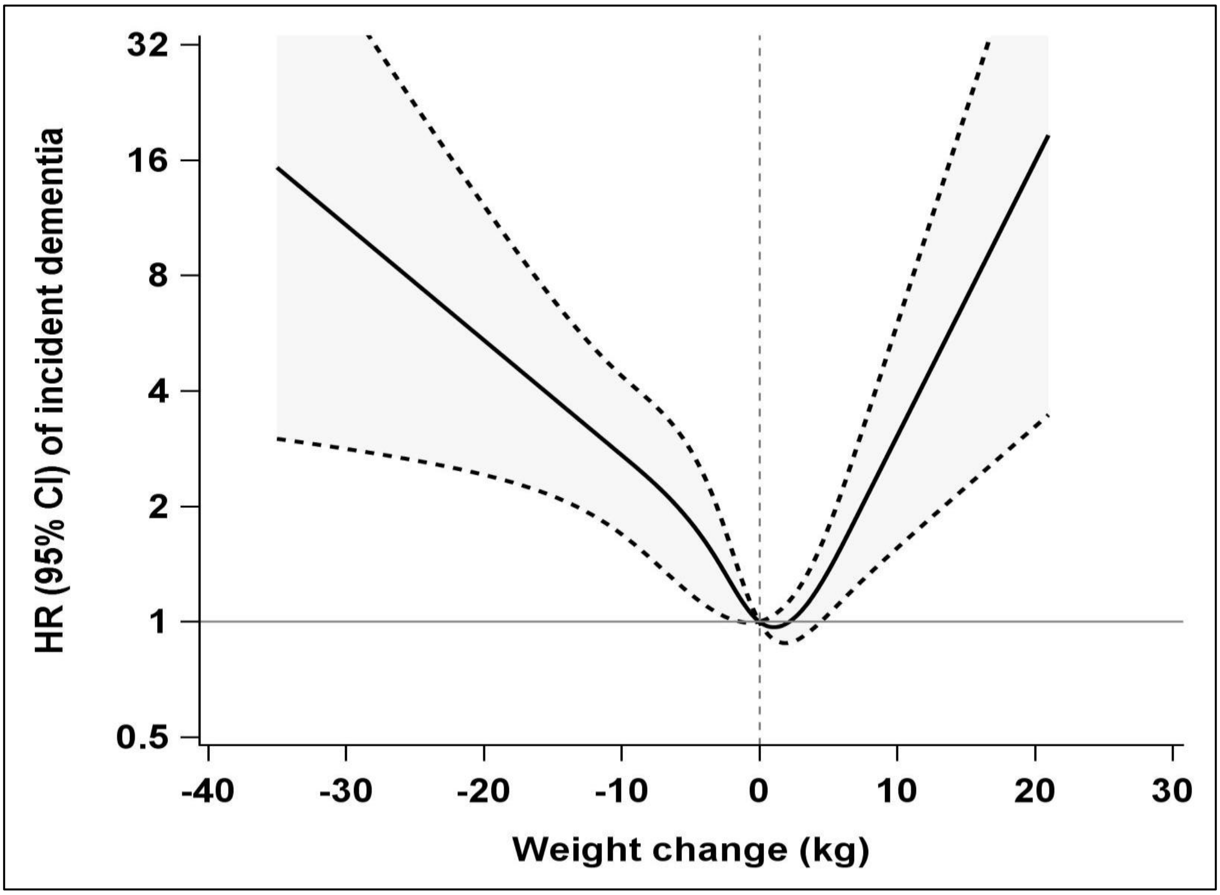

Supplementary Figure 2. Associations of weight change over 6 years with the risk of incident dementia.

Hazard ratios (HRs) and 95% confidence intervals (CIs) were calculated using multivariable Cox regression models with restricted cubic splines. Risk estimates were adjusted for baseline age, sex, education, smoking status, alcohol consumption, physical activity, medical history of cardiovascular diseases (ischemic heart disease, heart failure, atrial fibrillation, cerebrovascular disease, other cardiovascular diseases), hypertension, diabetes, depression, *APOE* ε4, and body mass index at baseline. *P* values for overall association and *P* values for non-linear association were both <.05.

Supplementary Table 4. Hazard ratios (HRs) and 95% confidence intervals (CIs) of the association of weight change over 6 years with dementia risk.

Weight change [c]	No. of subjects	No. of events/person-years	HR (95% CI)	
			Basic-adjusted [a]	Multi-adjusted [b]
**Continuous weight change**				
Loss <0 kg, per 1-unit decrease	921	71/4584	**1.09 (1.04−1.13)**	**1.08 (1.03−1.13)**
Gain >0 kg, per 1-unit increase	577	23/3125	**1.16 (1.04−1.28)**	**1.18 (1.04−1.32)**
**Categorical weight change**				
Large loss (>7.5 kg)	180	26/795	**2.93 (1.70−5.01)**	**2.92 (1.67−5.07)**
Moderate loss (2.5−7.5 kg)	429	29/2166	1.30 (0.78−2.17)	1.26 (0.75−2.10)
Stable (change ≤2.5 kg)	752	33/3980	Reference	Reference
Moderate gain (2.5−7.5 kg)	241	7/1336	0.72 (0.29−1.54)	0.75 (0.30−1.62)
Large gain (>7.5 kg)	71	7/363	**2.57 (1.03−5.59)**	**2.95 (1.16−6.53)**

Notes: [a] Adjusted for age, sex, and education.

[b] Additionally adjusted for smoking status, alcohol consumption, physical activity, hypertension, cardiovascular diseases, diabetes, depression, *APOE* ε4, and BMI at baseline.

[c] Weight change = $\text{Weight}_{\text{6th year follow-up}}$ - $\text{Weight}_{\text{baseline}}$.

Supplementary Table 5. Hazard ratios (HR) with 95% confidence intervals (CI) of incident dementia for weight change over 6 years by baseline body mass index (BMI).

**Baseline BMI**	**Weight change, HR (95% CI)**					***P* for interaction**
	**Large loss (> 7.5 kg)**	**Moderate loss (2.5-7.5 kg)**	**Stable (≤ 2.5 kg)**	**Moderate gain (2.5-7.5 kg)**	**Large gain (> 7.5 kg)**	
< 25 kg/m^2	2.45 (1.06 to 5.43)	0.75 (0.33 to 1.64)	Reference	0.87 (0.28 to 2.30)	1.75 (0.25 to 7.19)	0.334
≥ 25 kg/m^2	4.41 (2.00 to 9.78)	1.96 (0.93 to 4.19)	Reference	0.33 (0.05 to 1.31)	5.25 (1.63 to 14.6)	

Notes: Adjusted for age, sex, education, smoking status, alcohol consumption, physical activity, hypertension, cardiovascular diseases, diabetes, depression, *ApoE e4* genotype.

Weight change	*APOE ε4*	HR (95%CI)
Large loss (>7.5 kg)	No	3.84 (1.89 to 7.83)
	Yes	5.60 (2.20 to 13.2)
Moderate loss (2.5 to 7.5 kg)	No	1.30 (0.62 to 2.66)
	Yes	4.00 (1.91 to 8.27)
Stable (<=2.5 kg)	No	Reference
	Yes	3.39 (1.66 to 6.91)
Moderate gain (2.5 to 7.5 kg)	No	0.69 (0.20 to 1.90)
	Yes	2.90 (0.66 to 9.02)
Large gain (>7.5 kg)	No	2.54 (0.58 to 7.86)
	Yes	10.1 (2.79 to 29.3)

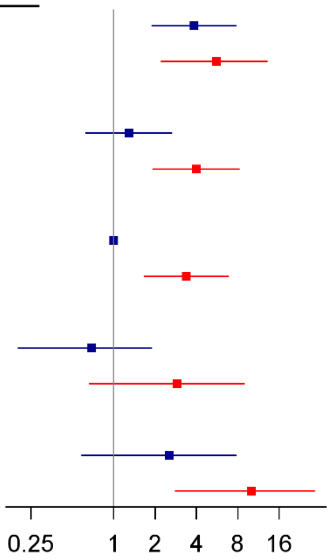

Supplementary Figure 3. Hazard ratios (HRs) and 95% confidence intervals (CIs) of the joint effect of *APOE* ε4 and weight change on incident dementia.

Adjusted for age, sex, education, smoking status, alcohol consumption, physical activity, medical history of cardiovascular diseases, hypertension, diabetes, depression, and body mass index at baseline.

Supplementary Table 6. Hazard ratios (HR) with 95% confidence intervals (CI) of incident dementia for body mass index (BMI) change by *APOE* genotype.

***APOE* genotype**	**HR (95% CI)**				
	**Large loss (>10%)**	**Moderate loss (5−10%)**	**Stable (≤5%)**	**Moderate gain (5−10%)**	**Large gain (>10%)**
APOE ε4 non-carriers	3.31 (1.62 to 6.67)	1.25 (0.55 to 2.64)	Reference	0.69 (0.16 to 2.07)	1.60 (0.25 to 5.81)
APOE ε4 carriers	3.07 (1.25 to 7.04)	1.19 (0.49 to 2.70)	Reference	0.87 (0.13 to 3.25)	3.74 (1.24 to 10.0)

Notes: Adjusted for age, sex, education, smoking status, alcohol consumption, physical activity, hypertension, cardiovascular diseases, diabetes, and depression. The multiplicative interaction between BMI change and *APOE* genotype is not statistically significant (P = .710).

Supplementary Table 7. Hazard ratios (HR) with 95% confidence intervals (CI) of incident dementia for weight change by *APOE* genotype.

***APOE* genotype**	**HR (95% CI)**				
	**Large loss (> 7.5 kg)**	**Moderate loss (2.5-7.5 kg)**	**Stable (≤ 2.5 kg)**	**Moderate gain (2.5-7.5 kg)**	**Large gain (> 7.5 kg)**
APOE ε4 non-carriers	4.00 (1.89 to 8.50)	1.46 (0.69 to 3.07)	Reference	0.72 (0.20 to 2.04)	2.70 (0.60 to 8.74)
APOE ε4 carriers	2.10 (0.81 to 5.05)	1.22 (0.56 to 2.59)	Reference	0.73 (0.17 to 2.32)	3.07 (0.81 to 9.37)

Notes: Adjusted for age, sex, education, smoking status, alcohol consumption, physical activity, hypertension, cardiovascular diseases, diabetes, depression, and baseline body mass index. The multiplicative interaction between weight change and *APOE* genotype is not statistically significant (P = .630).

Supplementary Table 8. Hazard ratios (HR) and 95% confidence intervals (CI) of incident Alzheimer's disease (AD), vascular dementia (VaD) according to BMI change and weight change over 6 years.

Body weight change	No. of subjects	AD		VaD	
		No. events/person-years	HR (95% CI)	No. events/person-years	HR (95% CI)
**BMI change**					
Large loss (>10%)	180	16/766	**2.04 (1.04 to 3.85)**	7/778	**17.0 (4.08 to 89.5)**
Moderate loss (>5%, ≤10%)	263	10/1,377	0.71 (0.32 to 1.41)	6/1,372	**5.79 (1.44 to 28.8)**
Stable (≤5%)	972	33/5,132	Reference	3/5,169	Reference
Moderate gain (>5%, ≤10%)	173	3/954	0.66 (0.15 to 1.90)	1/954	4.86 (0.23 to 43.9)
Large gain (>10%)	85	6/433	2.38 (0.84 to 5.82)	2/433	**9.70 (1.04 to 80.4)**
**Weight change**					
Large loss (>7.5 kg)	180	15/801	**2.29 (1.13 to 4.51)**	7/816	**16.5 (3.47 to 121.3)**
Moderate loss (>2.5, ≤7.5 kg)	429	18/2,173	1.04 (0.55 to 1.93)	7/2,178	**6.40 (1.42 to 45.6)**
Stable (≤2.5 kg)	752	25/3,988	Reference	2/4,012	Reference
Moderate gain (>2.5, ≤7.5 kg)	241	6/1,336	0.93 (0.34 to 2.18)	1/1,336	3.33 (0.15 to 38.4)
Large gain (>7.5 kg)	71	4/365	2.53 (0.71 to 7.00)	2/365	**12.2 (1.22 to 123.0)**

Notes: Adjusted for age, sex, education, smoking status, alcohol consumption, physical activity, medical history of cardiovascular diseases (ischemic heart disease, heart failure, atrial fibrillation, cerebrovascular disease, other cardiovascular diseases), hypertension, diabetes, depression, *APOE* genotype, and baseline BMI (only for weight change analysis).

Supplementary Table 9. Mixed effect model's β coefficients and 95% confidence interval (CI) for the annual Mini-Mental State Examination (MMSE) change related to BMI/weight change within the initial 6 years follow-up.

Body weight change	Basic-adjusted model [a]		Multi-adjusted model [b]	
	**β (95% CI)**	***P* value**	**β (95% CI)**	***P* value**
**BMI change**				
Large loss (>10%)	**-0.11 (-0.16 to -0.06)**	<0.001	**-0.09 (-0.14 to -0.04)**	<0.001
Moderate loss (>5%, ≤10%)	-0.03 (-0.07 to 0.02)	0.242	-0.03 (-0.07 to 0.01)	0.125
Stable (≤5%)	Reference		Reference	
Moderate gain (>5%, ≤10%)	0.01 (-0.04 to 0.06)	0.824	0.00 (-0.05 to 0.05)	0.953
Large gain (>10%)	**-0.07 (-0.14 to 0.00)**	0.046	-0.06 (-0.13 to 0.00)	0.062
**Weight change**				
Large loss (>7.5 kg)	**-0.11 (-0.16 to -0.06)**	<0.001	**-0.10 (-0.15 to -0.05)**	<0.001
Moderate loss (>2.5, ≤7.5 kg)	**-0.04 (-0.07 to 0.00)**	0.047	**-0.04 (-0.08 to -0.01)**	0.023
Stable (≤2.5 kg)	Reference		Reference	
Moderate gain (>2.5, ≤7.5 kg)	-0.03 (-0.07 to 0.02)	0.228	-0.03 (-0.07 to 0.01)	0.169
Large gain (>7.5 kg)	**-0.09 (-0.17 to -0.02)**	0.015	**-0.09 (-0.16 to -0.02)**	0.018

Notes: [a] Adjusted for age, sex, education, and their interaction terms with follow-up time.

[b] Adjusted for age, sex, education, smoking status, alcohol consumption, physical activity, hypertension, cardiovascular diseases, diabetes, depression, *APOE* ε4, and BMI at baseline (only for weight change analysis), and their interaction terms with follow-up time.

Supplementary Table 10. Hazard ratios (HR) with 95% confidence intervals (CI) of incident dementia according to BMI change and weight change excluding participants with cerebrovascular disease.

Body weight change	No. of subjects	HR (95% CI)[a]	HR (95% CI)[b]
**BMI change**			
Large loss (>10%)	170	**2.78 (1.60 to 4.75)**	**2.62 (1.48 to 4.53)**
Moderate loss (>5%, ≤10%)	255	1.06 (0.58 to 1.85)	0.87 (0.47 to 1.54)
Stable (≤5%)	933	Reference	Reference
Moderate gain (>5%, ≤10%)	167	0.76 (0.26 to 1.75)	0.78 (0.27 to 1.83)
Large gain (>10%)	81	2.27 (0.91 to 4.84)	2.06 (0.81 to 4.60)
**Weight change**			
Large loss (>7.5 kg)	168	**2.65 (1.50 to 4.63)**	**2.55 (1.40 to 4.57)**
Moderate loss (>2.5, ≤7.5 kg)	410	1.13 (0.66 to 1.92)	1.05 (0.61 to 1.79)
Stable (≤2.5 kg)	728	Reference	Reference
Moderate gain (>2.5, ≤7.5 kg)	232	0.65 (0.24 to 1.45)	0.65 (0.24 to 1.46)
Large gain (>7.5 kg)	68	**2.60 (1.03 to 5.68)**	**2.90 (1.13 to 6.53)**

Notes: [a] Adjusted for age, sex, and education.

[b] Additionally adjusted for smoking status, alcohol consumption, physical activity, hypertension, cardiovascular diseases (except cerebrovascular disease), diabetes, depression, *APOE* ε4, and BMI at baseline (only for weight change analysis).

Supplementary Table 11. Hazard ratios (HR) and 95% confidence intervals (CI) of the association of BMI/weight change with dementia risk using datasets with imputed BMI or weight.

**Body weight change**	**No. of subjects**	**No. of events/person-years**	**HR (95% CI) [a]**
BMI change [b]			
Large loss (>10%)	189	28/777	2.91 (1.74 to 4.77)
Moderate loss (5−10%)	267	19/1384	1.01 (0.57 to 1.71)
Stable (≤5%)	1006	47/5237	Reference
Moderate gain (5−10%)	177	5/972	0.76 (0.26 to 1.76)
Large gain (>10%)	89	9/447	2.78 (1.22 to 5.72)
Weight change [c]			
Large loss (>7.5 kg)	187	27/814	2.81 (1.63 to 4.80)
Moderate loss (2.5−7.5 kg)	440	30/2193	1.25 (0.75 to 2.07)
Stable (change ≤2.5 kg)	777	35/4062	Reference
Moderate gain (2.5−7.5 kg)	252	9/1379	0.89 (0.40 to 1.80)
Large gain (>7.5 kg)	72	7/369	2.94 (1.16 to 6.48)

Notes: [a] Adjusted for age, sex, education, smoking status, alcohol consumption, physical activity, hypertension, cardiovascular diseases, diabetes, depression, *APOE* ε4, and BMI at baseline (only for weight change analysis).

[c] Percent change in BMI = [BMI $_{\text{6th year follow-up}}$ - BMI $_{\text{baseline}}$] / BMI $_{\text{baseline}}$.

[d] Weight change = Weight $_{\text{6th year follow-up}}$ - Weight $_{\text{baseline}}$.

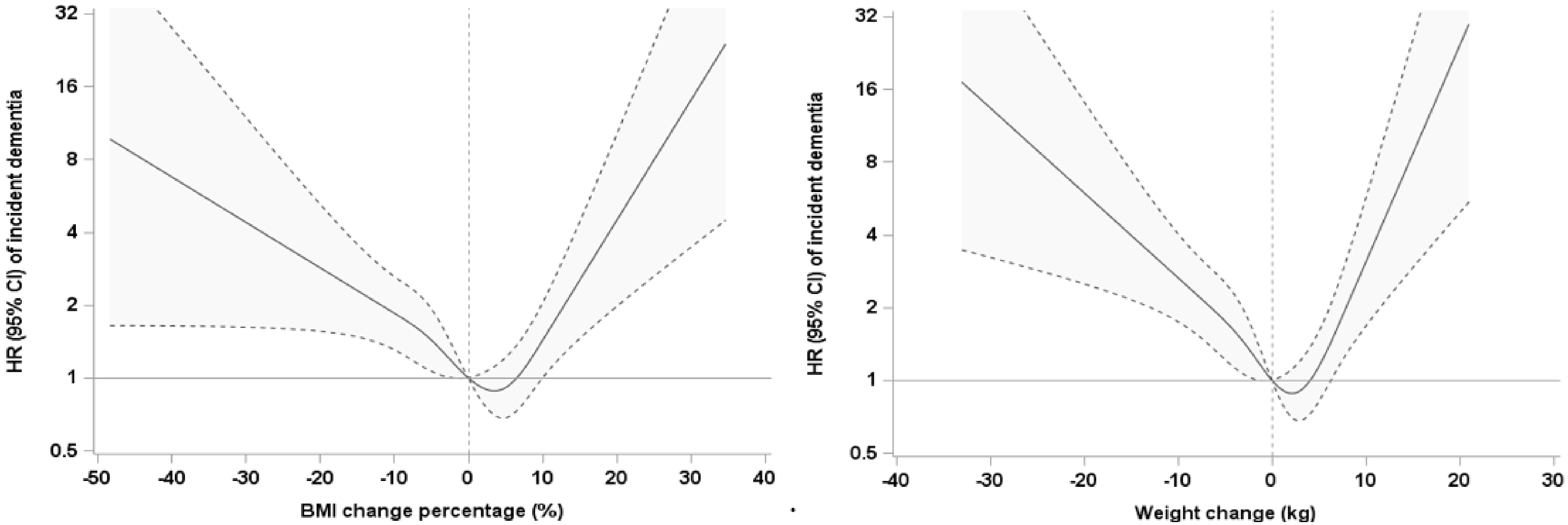

Supplementary Figure 4. Associations of body mass index (BMI) and weight change with risk of incident dementia

The associations of continuous BMI/weight change with subsequent dementia were modelled using restricted cubic splines with four knots at the at the value of -10%, -5%, 5%, and 10% for BMI change percentage and -7.5 kg, -2.5 kg, 2.5 kg, and 7.5 kg for weight change. Risk estimates were adjusted for baseline age, sex, education, smoking status, alcohol consumption, physical activity, medical history of cardiovascular diseases (ischemic heart disease, heart failure, atrial fibrillation, cerebrovascular disease, other cardiovascular diseases), hypertension, diabetes, depression, *APOE* ε4, and BMI at baseline (only for weight change analysis). *P* values for overall association and *P* values for non-linear association were both <.05.
